# Pregnancy outcome in women with Eisenmenger’s syndrome: a case series from west China

**DOI:** 10.1186/s12884-016-1153-z

**Published:** 2016-11-16

**Authors:** Ruiqi Duan, Xiumei Xu, Xiaodong Wang, Haiyan Yu, Yong You, Xinghui Liu, Aiyun Xing, Rong Zhou, Mingrong Xi

**Affiliations:** 1 Department of Obstetrics and Gynecology, West China Second University Hospital, Sichuan University, No. 20, 3rd section, South Renmin Road, Chengdu, 610041 China; 2 ICU of Gynecology & Obstetrics, West China Second University Hospital, Sichuan University, No. 20, 3rd section, South Renmin Road, Chengdu, 610041 China; 3 Key Laboratory of Birth Defects and Related Diseases of Women and Children (Sichuan University), Ministry of Education, No. 20, 3rd section, South Renmin Road, Chengdu, 610041 China

**Keywords:** Pregnancy, Eisenmenger’s syndrome (ES), Pulmonary arterial hypertension (PAH), Maternal outcome, Fetal outcome

## Abstract

**Background:**

Eisenmenger’s syndrome (ES) consists of pulmonary hypertension with a reversed or bidirectional shunt at the atrioventricular, or aortopulmonary level.

The cardiovascular changes that occur during the pregnancy contribute to the high maternal morbidity and mortality in patients with ES. This study is to assess maternal and fetal outcomes in patients with ES.

**Methods:**

This study is a retrospective analysis of 11 pregnancies in women with ES who delivered at a tertiary care center in west China between 2010 and 2014. Cases were divided into group I (maternal survival) and group II (maternal death). Clinical data were noted and analyzed.

**Results:**

All ES patients presented with severe pulmonary arterial hypertension (PAH). Four maternal deaths were recorded (maternal mortality of 36%). Only one pregnancy continued to term. Ventricular septal defect diameter in group II was larger than that in group I (2.93 ± 0.76 cm vs. 1.90 ± 0.54 cm, *p* < 0.05). Arterial oxygen saturation and pre-delivery arterial oxygen tension during oxygen inhalation were significantly lower in group II (*p* < 0.05). Pulmonary arterial blood pressure (PABP) in both groups were high while ejection fractions (EF) were significantly lower in group II (*p* < 0.05). The incidence of pre-delivery heart failure in group II was substantially higher than in survivors (100 vs.14.3%, *p* < 0.05). Fetal complications were exceptionally high: preterm delivery (88%), small for gestational age (83%), fetal mortality (27%) and neonatal mortality (25%).

**Conclusions:**

In west China,the perinatal outcome of pregnant women with ES is poor, especially when complicated with high pulmonary arterial hypertension (PAH). Pregnancy remains strongly contraindicated in ES. Effective contraception is essential, and the option of terminating pregnancy in the first trimester should be presented to pregnant women with ES.

## Background

Eisenmenger’s syndrome (ES) consists of pulmonary hypertension with a reversed or bidirectional shunt at the atrioventricular, or aortopulmonary level. It was first described in 1897 by Victor Eisenmenger in a single patient and in 1958 Paul Wood provided further data in a large patient population with congenital heart defects [1]. Congenital heart defects that may result in Eisenmenger’s syndrome include ventricular septal defect (VSD), atrioventricular septal defect (AVSD) or atrioventricular canal defect (AVCD), patent ductusarteriosus (PDA), atrial septal defect (ASD), D-transposition of the great vessels, and surgically created aortopulmonary connections. The likelihood of developing ES depends on the size and location of the intracardiac defect [2]. ES gradually leads to progressive right ventricular (RV) failure with digital clubbing, cyanosis, dyspnea, edema, a loud pulmonary component of second heart sound on auscultation, right ventricular hypertrophy in electrocardiography, and arrhythmias.

Although ES progresses slowly, patients have a poor quality of life. The increased circulatory burden in gravid patients contributes to high maternal mortality rates (30–50%) in pregnancy in ES [3–7]. The major causes of death in ES are right ventricular failure, pulmonary hypertension crisis, arrhythmia, and stroke. Despite modern developments in obstetric medicine and cardiovascular interventions, the prognosis of pregnancy with ES has not improved greatly, and this is particularly so in low income countries. ES is class IV in the WHO pregnancy risk classes and is regarded as contraindication for pregnancy [8]. Meanwhile, fetal outcomes are also poor, with a high risk of spontaneous abortion, intrauterine growth restriction (IUGR), preterm birth, low-birth weight and congenital cardiac malformations [9].

However, some women with ES do conceive. Termination of pregnancy is generally recommended for those who present early in the pregnancy. There are few reports of ES in pregnancy from high income countries and less from low income countries, especially studies examining with aggressive pulmonary arterial hypertension (PAH). The present study reviews perinatal outcomes and management of women with ES in pregnancy with aggressive PAH in west China.

## Methods

This was a retrospective study. Approval was obtained from the Institutional Review Board of West China Second University Hospital. Medical records were reviewed of pregnant women with congenital heart disease (CHD) who were admitted during 2010 to 2014 to West China Second University Hospital, the regional tertiary referral center.

### Subjects

Eleven pregnant women with ES admitted to the hospital were identified in CHD pregnants. The majority of the patients had been transferred from other local hospitals. We received written permission from the patient or relatives to use the medical records, the images and the patients’ information. Cases were retrospectively divided into two groups, group I (maternal survival, 7 cases) and group II (maternal death, 4 cases).

### Management of ES

After admission, patient evaluation was performed by a multidisciplinary team, consisting of obstetrician, midwife, cardiologist, anesthetist, intensive care physician and neonatologist. Complete general physical, cardiovascular, and obstetric examination were performed. New York Heart Association (NYHA) class was assigned. Laboratory investigations were undertaken.

Bedrest and oxygen therapy were provided. Oxygen was administered by mask at 5 L/min. A variety of diuretics were used if necessary. The status of the mother and fetus was closely monitored. Patients were evaluated by obstetricians, cardiologist and intensive care physician daily. Ultrasound scans and cardiotocography (CTG) were performed daily to evaluate fetal status. Worsening of maternal clinical condition, labor or fetal distress were indications for delivery. Arterial, central venous, and/or pulmonary artery monitoring were performed to optimally manage the mother. Hemodynamic measurements and blood gas analyses were carried out in all cases upon admission, during treatment, and after delivery. Antibiotic prophylaxis was provided for endocarditis prophylaxis.

### Data collection

Maternal baseline data reviewed included age, parity, preconception body weight and height, place of residence, education level, nature of underlying cardiac lesion, treatment and cardiac surgery before pregnancy, and results of routine examination during pregnancy. Cardiac functional class and heart failure category (American NYHA) after admission were noted. Cardiac lesion size, ejection fraction (EF), reversed or bidirectional shunt at the atrioventricular or aortopulmonary level, and pulmonary arterial blood pressure (PABP) were acquired from echocardiography and Doppler cardiographic examination upon admission. Arterial oxygen saturation, arterial oxygen tension and hemoglobin (Hb) were noted upon admission. Mode of delivery, anesthesia options, operative time and blood loss were also reviewed.

Fetal parameters including birth weight, Apgar score, fetal death or neonatal death were studied.

### Statistical analyses

Parameters were analyzed using Chi-squared test, Fisher’s exact test, two-tailed student’s *t*-test and Mann-Whitney *U* test. *P*- value < 0.05 was considered statistically significant. Analysis was performed with SPSS version 17.0.

## Results

### Maternal characteristics

Subjects were 11 pregnant patients who were diagnosed with ES. All cases were singleton pregnancies. Mean maternal age was 25.7 years (range: 17–35 years). Mean weeks of gestation at admission was 32^+5^ weeks (range: 21–39^+1^ weeks). Pre-conception body height and weight were 156.5 cm (range: 150–166 cm) and 57.5 kg (range: 46–69 kg). In group I (surviving mothers), six patients (out of seven) had history of prior pregnancy, with two live births. Three out of the four patients in group II had a history of prior pregnancy and delivery. The incidence of prior delivery history of group II was significantly higher than that of group I (*p* < 0.05).

CHD in three cases (two in group I and one in group II) were not identified until admission to the local hospital. Among the eleven cases, eight patients (five in group I, and three in group II) knew about their CHD but refused to visit the cardiologist regularly, and refused pre-conception counseling by obstetrician and cardiologist, and there was no pre-pregnancy hemodynamic data. Among eight patients, only two patients (city residents) received regular cardiologist and obstetric care during pregnancy, and both refused abortion despite of knowledge about the risks and prognosis. Nine patients had no regular care,all derived from the rural area. None of the patients had undergone cardiac surgery before pregnancy. In regard to education level, one patient graduated from junior college, one was illiterate, and the others had finished middle school or primary school. Maternal characteristics of all patients in the study are shown in Table 1.

**Table 1:** Maternal characteristics

Case	Age (years)	Height (cm)	Weight (kg)	Residence	Education level	Regular care	Cardiac surgery	GA at admission	Age of patient when CHD diagnosed
1	28	166	59	Rural	Middle school	N	N	36^+4^	8 years old
2	29	160	50	Rural	Middle school	N	N	21	9 gestational weeks
3	29	160	69	Rural	Illiterate	N	N	29^+5^	18 years old
4	28	150	46	Rural	Primary school	N	N	30^+2^	29^+3^ gestational weeks
5	21	155	66	Rural	Middle school	N	N	39^+1^	1 year old
6	25	157	64	Urban	Junior college	Y	N	35^+5^	10 years old
7	20	152	53.5	Urban	Middle school	Y	N	29^+5^	1 year old
**8**	**17**	**160**	**55**	**Rural**	**Middle school**	**N**	**N**	**34** ^**+2**^	**3 years old**
**9**	**35**	**155**	**63.5**	**Rural**	**Primary school**	**N**	**N**	**36**	**35** ^**+5**^ **gestational weeks**
**10**	**25**	**150**	**55**	**Rural**	**Middle school**	**N**	**N**	**34**	**19 years old**
**11**	**26**	**156**	**51**	**Rural**	**Primary school**	**N**	**N**	**33** ^**+4**^	**2 years old**

*GA* gestational weeks, *CHD* congenital heart defect. Boldface: Group II patients (maternal death)

### Cardiac characteristics

In group I, five cases had VSD and two cases ASD; one patient had heart failure (HF). During admission, mean pulmonary arterial blood pressure (PABP) was 102 ± 27 mmHg (range: 77–139 mmHg); mean ejection fraction (EF) was 64 ± 1.3% (range: 62–66%); mean arterial oxygen saturation and tension under oxygen inhalation was 80 ± 6% (range: 74–90%), and 46.6 ± 3.0 mmHg (range: 43–51 mmHg) respectively; mean hemoglobin was 131 ± 11.6 g/L (range: 119–150 g/L).

In group II, all cases had VSD with HF. Upon admission, mean PABP was 101 ± 22 mmHg (range: 78–128 mmHg); mean EF 51 ± 11% (range: 35–60%); mean arterial oxygen saturation and tension under oxygen inhalation was 63 ± 3% (range: 60–67%) and 40 ± 1.4 mmHg (range: 38–41 mmHg) and mean hemoglobin was 124 ± 11 g/L (range: 107–130 g/L). Detailed information is shown in Table 2.

**Table 2:** Maternal cardiac characteristics

Case	Cardiac lesion	Lesion diameter (cm)	EF (%)	PABP (mmHg)	Arterial oxygen saturation (%)	Arterial oxygen tension (mmHg)	Hb (g/l)
1	VSD	1.4	64	100	90	51	150
2	ASD*	3.0	62	95	76	46	140
3	VSD	1.9	63	84	79	47	124
4	VSD	1.8	64	139	74	45	137
5	ASD	2.1	66	77	87	50	125
6	VSD	1.6	65	139	78	44	119
7	VSD	1.5	63	78	75	43	121
**8**	**VSD**	**2.9**	**57**	**88**	**64**	**38**	**107**
**9**	**VSD**	**2.5**	**52**	**110**	**67**	**41**	**128**
**10**	**VSD**	**4.0**	**35**	**128**	**62**	**41**	**129**
**11**	**VSD**	**2.3**	**60**	**78**	**60**	**40**	**130**

*VSD* ventricular septal defect, *ASD* atrial septal defect, ASD* ASD with patent ductus arteriosus, *Hb* hemoglobin, *EF* ejection fraction. Boldface: Group II patients (maternal death)

In this study, 9/11 patients had VSD. The cardiac lesion diameter of VSD in group II was significantly larger than that in group I (2.93 ± 0.76 cm vs. 1.9 ± 0.54 cm, *p* < 0.05). The incidence of HF in group II was significantly higher than that in group I (100 vs. 14.3%, *p* < 0.05); arterial oxygen saturation and arterial oxygen tension under oxygen inhalation were significantly lower in group II (*p* < 0.05). Meanwhile, we found that PABP in both groups were high, and EF were significantly lower in group II (*p* = 0.008). Clinical cardiac data are shown in Table 2.

### Complications

In group I, after delivery, one patient had pulmonary infection (PI) and one patient had severe preeclampsia (SP). In group II, three patients had PI, two patients had SP. The tendency to PI and hypertensive disorders in pregnancy were higher in group II, although not reaching statistical significance (Table 3).

**Table 3:** Maternal clinical data

Case	Maternal complications	History of pregnancy	Maternal Death
1	HF, ICP, PI	1st pregnancy: abortion; 2nd pregnancy: abortion	N
2	–	1st pregnancy: abortion; 2nd pregnancy: CS for massive hemorrhage during labor induction 3 years earlier with IUGR and oligohydramnios; neonatal demise	N
3	PE after anticoagulation (4 months ago)	1st pregnancy: CS 11 years earlier with birth weight 3250 g and healthy baby 2nd pregnancy: abortion	N
4	–	1st pregnancy: premature delivery 6 years earlier, birth weight 2400 g healthy 2nd pregnancy: abortion	N
5	PA, oligohydramnios	1st pregnancy: abortion	N
6	left thoracic deformity	1st pregnancy: abortion	N
7	SP, thrombocytopenia	none	N
8	HF, SP, PI	none	Y
9	HF, SP	1st pregnancy: term vaginal delivery 9 years earlier and baby healthy till now	Y
10	HF, PI	1st pregnancy: vaginal delivery 4 years earlier and baby died (prematurity)	Y
11	HF, PI	1st pregnancy: CS 7 years earlier with birth weight 2500 g, baby alive 2nd pregnancy: induction at 17 gestational weeks 6 years earlier 3rd pregnancy: abortion 4th pregnancy: abortion	Y

*ICP* intrahepatic cholestasis of pregnancy, *PI* pulmonary infection, *HF* heart failure, *SP* severe preeclampsia, *PA* placental adhesion, *PE* pulmonary embolism

### Clinical outcomes

Perinatal clinical data are shown in Table 4. There were four patients admitted for induction before 32 gestational weeks in group I: two because of fetal demise (FD) and one for fetal hydrocephalus. No case in group II delivered before 32 gestational weeks.

**Table 4:** Delivery data and fetal outcomes

Case	Mode of delivery	Operative time	Operative blood loss	GA at delivery	Anesthesia	Fetal outcome	Birth weight	Newborn outcome
1	CS	38 min	250 ml	36^+5^	Epidural	SGA	1740 g	live
2	Induction	/-	/	21	/	FD	370 g	/
3	CS	35 min	200 ml	29^+6^	General	SGA	920 g	dead
4	CS	60 min	300 ml	30^+3^	General	SGA	970 g	dead
5	CS	48 min	220 ml	39^+2^	Epidural	SGA	1825 g	live
6	CS	43 min	300 ml	36	General	SGA	2045 g	live
7	CS	55 min	200 ml	29^+5^	General	FD	680 g	/
**8**	**CS**	**42 min**	**270 ml**	**34**	**General**	**SGA**	**1690 g**	**live**
**9**	**CS**	**30 min**	**150 ml**	**34** ^**+2**^	**General**	**SGA**	**1630 g**	**live**
**10**	**DBD**	**/-**	**/-**	**36**	**/-**	**FD**	**/-**	**/**
**11**	**CS**	**28 min**	**260 ml**	**33** ^**+4**^	**Epidural**	**/**	**1720 g**	**live**

*DBD* maternal death before delivery, *FD* fetal demise, *SGA* small for gestational age; Boldface: Group II patients (maternal death)

In group I, six patients underwent CS and one patient had induced vaginal delivery (at 21 gestational weeks, for fetal demise). In group II, three patients underwent CS and one mother died before delivery. CS was performed under general anesthesia in six cases, three cases under epidural anesthesia. Mean operation time and measured blood loss at surgery did not significantly differ between the two groups. Details are shown in Table 4.

Four maternal deaths occurred in our study; overall mortality was 36.4% (4/11). Case #8 was diagnosed with VSD at three years of age following routine examination. Her family refused cardiac surgery and there was no regular follow-up by a cardiologist. Her cardiac condition progressed to ES two years before pregnancy. During this, her first, pregnancy, she was transferred to our hospital at 34^+2^ weeks for acute heart failure, with severe preeclampsia and thrombocytopenia. Her ECG showed sinus tachycardia with right axis deviation (+129^°^), suggesting bilateral atrial enlargement and ventricular hypertrophy. With HF treatment and preoperative preparation, emergency CS for fetal distress was performed on the admission day under general anesthesia. After delivery, she rapidly developed severe pulmonary infection and uncontrollable heart failure, and died one day after operation. Her baby is healthy.

Case #9 was diagnosed to have VSD just two days ago before admission. She had one term vaginal delivery nine years earlier, and that baby is alive without abnormal manifestations. She had no care in this current pregnancy and was admitted at 36 weeks with severe preeclampsia and heart failure. Her ECG showed sinus tachycardia with right bundle branch block and right axis deviation (+264^°^), suggesting right ventricular hypertrophy and bilateral atrial enlargement. She died before delivery on the day of admission, resulting from uncontrollable heart failure. Her fetus died in utero.

Cases #10 and #11 were similar to the 8th case, in that they had received no cardiac treatment or follow-up. Neither received cardiologist or obstetrician care during this pregnancy. Case #10 had one vaginal delivery four years earlier, in which the baby died due to prematurity. Case #11 had one CS seven years earlier (birth weight 2500 g) and that baby is alive without any abnormality. In addition, Case #11 had one induction six years earlier at 17 gestational weeks for FD, and two abortions. They were admitted into our hospital respectively at 34 and 33^+4^ gestational weeks for heart failure with severe PAH. Upon admission, they were both found to have large VSD’s and had progressed to ES. The ECG of Case #10 after admission showed sinus rhythm with left bundle branch block and left axis deviation (−62^°^), suggesting left atrial enlargement and right ventricular hypertrophy. The ECG of Case #11 showed sinus rhythm, complete right bundle branch block with first degree atrioventricular block, right ventricular hypertrophy and left atrial enlargement. Emergency CS under general anesthesia was performed on the day of admission in both patients because of labor. After delivery, to treat severe pulmonary infection and heart failure, both mothers were supported with ventilators. However, both died three days after operation. Post-mortem examination was refused. Their babies both survived without any abnormal manifestation.

No fetus had congenital heart defects in the study. Two fetal deaths occurred at 21 and 29^+5^ gestational weeks in group I and one fetal death at 36 gestational weeks due to maternal death in group II. There were eight live births in the study; seven preterm, and one term birth; two neonates died within one week after birth for prematurity,six babies survived until now. The incidence of fetal mortality, preterm delivery and neonatal mortality was 27.27% (3/11), 87.5% (7/8) and 25% (2/8) respectively. The incidence of small for gestational age (SGA) [10] was 83.33% (5/6) of the live babies, one with congenital hydrocephalus, five with no persistent abnormality until now. Fetal outcomes are presented in Table 4.

## Discussion

Pregnancy is well known to require significant hemodynamic changes in women to meet the rising metabolic demands of the mother and the fetus. The cardiovascular changes that occur during the pregnancy contribute to the high maternal morbidity in patients with Eisenmenger’s syndrome, including heart failure, dyspnea, syncope and sudden death. Therefore, ES is regarded as an absolute contraindication for pregnancy [8]. If a woman with ES chooses to continue with pregnancy, oxygen therapy, aggressive use of pulmonary vasodilator therapies, and care by a specialist multidisciplinary team may help to minimize mortality [11].

In this study, most patients with ES were rural residents in west China, with low educational level and poor physical status. They suffered from cardiac lesions over a long period with chronic tolerance to CHD, or no symptoms. Perhaps as a result of lack of general and special medical knowledge, these rural patients all refused cardiac surgery and were not followed up by cardiologists. Most of them were not sufficiently conscious of the increased risks and mortality of pregnancy with ES, and there was lack of pre-conception counseling nor regular care with the cardiologist and obstetrician during pregnancy. Although early pregnancy termination was recommended for patients with known ES, none of them chose to discontinue pregnancy in the first trimester. After 32 gestational weeks, the hemodynamic burden of pregnancy peaks, with the highest risk for maternal and fetal life, especially in the presence of a large VSD. In this study, five cases with heart failure presented at this stage, and four of those women eventually died with uncontrollable heart failure.

From this study, we found that patients with pulmonary infection and/or SP were more likely to succumb to heart failure and death. Such complications likely further exacerbate cardiovascular and pulmonary vascular demands. In our study, PABP in all patients was very high. Pulmonary hypertension is associated with high morbidity and mortality during pregnancy. For patients with aggressive PAH in pregnancy, hypertension reduction using sildenafil or prostacyclin analogs has not been the primary therapeutic regimen because of associated adverse effects [12]. However, Rao et al. deemed that for women with pulmonary hypertension who do become pregnant and do not wish to terminate pregnancy, it is important to continue pre-pregnancy management such as anticoagulation, prostacyclin analogs, and sildenafil [13]. Because of limited evidence with only a few published case series, there is no consensus on the use of such therapies for ES patients in pregnancy. In our hospital, sildenafil or prostacyclin analogs were not given to ES patients with aggressive PAH throughout pregnancy. Although large intrapulmonary thrombi occur in up to a quarter of patients with Eisenmenger syndrome, no evidence of thrombi was identified in our study and no anticoagulation was performed.

Close pregnancy monitoring and supportive care for these patients are essential, especially for cases with aggressive PAH. To monitor cardiac and hemodynamic status of ES patients, arterial line, central venous catheter or right-sided catheterization may be of use. However, invasive monitoring is not routinely recommended to pregnant women because of the increased risk of pulmonary artery rupture or thrombosis, unless the patient is unstable or with high-risk lesions. Therefore, monthly echocardiograms and weekly examination for these women are needed. If PABP aggravates during early trimester, the prognosis of continuing pregnancy may be particularly bad, and therapeutic abortion is recommended. If these patients insist on continuing pregnancy, hospitalization in the second trimester is highly recommended [14]. They should be admitted in a tertiary center, with a multidisciplinary team approach.

Due to few reports, it should be noted that the ideal mode of delivery in ES patients with PAH is controversial [7, 15]. Compared with cesarean delivery, although vaginal delivery is associated with less risk of haemorrhage, infection and venous thromboembolism, vaginal delivery is associated with increased basal cardiac output and increased output with every uterine contraction,which would promote cardiac arrhythmia and worsen heart failure. Furthermore, ES patients with PAH will be delivered early due to serious disease condition with unfavourable cervix and labour induction is likely to be difficult in these patients. Advantages of cesarean delivery include that time and opportunity of delivery can be controllable, and presence of senior staff can usually be ensured [16, 17]. Since our cases had ES with high PAH, we thought CS was suitable; our cases in third trimester were all delivered by cesarean section, except for one death before delivery. As far as blood loss and time of operation were concerned, no case lost more than 300 ml and all operations were completed within an hour. Because of the greater stability of hemodynamics, CS may be safer than vaginal delivery for these patients with severe baseline hemodynamic abnormalities.

In our study, 6/9 ES patients underwent CS under general anesthesia, whereas most of the previous reported ES patients were given epidural anesthesia or combined spinal epidural anesthesia [16, 18]. Either epidural or general anesthesia has been used with successful outcomes. Gurumurthy et al. reported general anesthesia in patient with ES undergoing cesarean section [19]. We hold to the same opinion as Gurumurthy’s: general anesthesia could allow for adequate control of pain and early initiation of thromboprophylaxis. Moreover, it allows for placement of an esophageal ultrasound probe after trachea cannulation for ultrasonic monitoring.

The presence of maternal cardiovascular disease places the fetus and neonate at greater risk. Increased rates of miscarriage, SGA, prematurity, intrauterine fetal demise, and neonatal death are present in many forms of maternal CHD whether repaired or unrepaired [20]. ES with PAH in pregnancy presents an increased risk of fetal complications such as stillbirth, SGA and preterm delivery [6]. Our study supports these findings and suggests that severe PAH in ES patients is likely to cause even higher risk. Our findings support that ES patients with severe PAH should be counseled regarding the discouraging outcomes and options for termination, especially in low income countries as reported by Lin et al. [21].

More women with ES are surviving in recent years because of advances in medicine and neonatal care. However, the prognosis for a pregnant woman with ES has not improved. Gleicher et al. reviewed 44 published cases and 52% pregnancy-associated mortality with ES, with 36.1% mortality on first pregnancy, 26.7% on second pregnancy and 33.3% on third pregnancy [4]. Our study indicated that the maternal mortality was 50% on first pregnancy, 50% on second pregnancy and the delivery frequency was higher in group II. Drenthen et al. reported that in pregnant ES patients, the occurrence of heart failure was 21%, prematurity rate 64.7%, SGA 37.5%, fetal mortality 9.5 %, and death within the first year of life 18.2% [22]. Stoddart et al. reviewed 30 cases and found that maternal mortality was 39%; the incidence of preterm deliveries, perinatal death, and total fetal wastage was respectively 44, 14 and 44% [23]. Alvira et al. reported maternal mortality was 33% according to a prospective study of 12 cases [5]. Yentis et al. studied 15 cases and identified that maternal mortality, fetal mortality and prematurity rate was respectively 40, 8 and 85% [6]. In our study, the incidence of maternal HF, maternal mortality, preterm birth, SGA, fetal mortality and neonatal mortality were respectively 45, 36, 88, 83, 27 and 25% respectively. Our maternal outcomes were similar to the other reports, whereas fetal outcome was worse than other reports. Most of our cases were from the rural areas of west China where there is a less developed medical system, and rejection of regular prenatal care with obstetricians and cardiologists is common. The development of SP may be an additional risk factor in ES. Phupong et al. recently performed a small review of five cases of ES with preeclampsia, and found that maternal mortality and premature delivery rate were respectively 80 and 60% [24], substantially higher than those without SP. Wang and Liu presented four cases of pregnancy complicated with HELLP syndrome in the setting of ES: three of the mothers died [25]. In our study, the incidence of SP in group II (maternal death) was higher than group I. It seems highly likely that SP or HELLP may aggravate the dangers of pregnancy in ES.

There have been few reports suggesting particular success in pregnancy with ES. Wang et al. recently reported perinatal outcome in 13 cases with ES and found maternal mortalityto be 7.7% (1/13), while infant loss was 38.5% and prematurity rate was 100%. The only maternal death occurred 17 days after delivery etc., resulting from HELLP syndrome, lung infection and heart failure [16]. However, optimistic reports of maternal survival rates are rare. We think in Wang’s study the lower maternal mortality may result from the higher delivery rate before 28 weeks (38.5%; 5/13). The progressive hemodynamic changes from second trimester to the third trimester add to the burden of a compromised right ventricle. Further studies with more cases will be needed to confirm this. In our study, 76.9% (10/11) cases delivered beyond 28 weeks’gestation and the maternal mortality rate was 36%, similar to other reports.

## Conclusion

In conclusion, the present study examined perinatal outcomes and management of women with ES in pregnancy with aggressive PAH in west China. Timely diagnosis and treatment of these patients’ underlying cardiac lesion in early childhood are critical for their pregnancy and delivery. Pregnancy in ES, especially complicated with aggressive PAH, is still associated with high maternal and fetal morbidity and mortality. Effective preconception counseling is essential, and termination of pregnancy in first trimester advisable. Risks will increase significantly in pregnancy continued into the third trimester. If the ES patient with PAH wishes to continue pregnancy, they should be monitored closely and managed in a tertiary center with collaborative efforts among obstetricians, cardiologists, anesthesiologists, pediatricians and intensivists. There is no standardized approach to management of ES in pregnancy; successful perinatal outcomes seem heavily dependent on the individualization of treatment for each patient.

## Abbreviations

ASD: Atrial septal defect
CHD: Congenital heart disease
EF: Ejection fraction
ES: Eisenmenger’s syndrome
FD: Fetal demise
HF: Heart failure
IUGR: Intrauterine growth retardation
NYHA: New York Heart Association
PABP: Pulmonary arterial blood pressure
PAH: Pulmonary arterial hypertension
PDA: Patent ductus arteriosus
PI: Pulmonary infection
RV: Right ventricular
SGA: Small for gestational age
SP: Preeclampsia
VSD: Ventricular septal defect

## References

[1] Wood P (1958). The Eisenmenger syndrome or pulmonary hypertension with reversed central shunt. Br Med J.

[2] Beghetti M, Galie’ N (2009). Eisenmenger syndrome a clinical perspective in a new herapeutic era of pulmonary arterial hypertension in adults. J Am Coll Cardiol.

[3] Jones AM, Howitt G (1965). Eisenmenger syndrome in pregnancy. Br Med J.

[4] Gleicher N, Midwall J, Hochberger D, Jaffin H (1979). Eisenmenger’s syndrome and pregnancy. Obstet Gynecol Survey.

[5] Avila WS, Grinberg R, Snitcowsky R, Faccioli R, Da Luz PL, Bellotti G, Pileggi F (1995). Maternal and fetal outcome in pregnant women with Eisenmenger’s syndrome. Eur Heart J.

[6] Yentis SM, Steer PJ, Plaat F (1998). Eisenmenger’s syndrome in pregnancy: maternal and fetal mortality in the 1990s. Br J Obstet Gynaecol.

[7] Weiss BM, Zemp L, Seifert B, Hess OM (1998). Outcome of pulmonary vascular disease in pregnancy: systemic overview from1978 through 1996. J Am Coll Cardioll.

[8] Thorne S, MacGregor A, Nelson-Piercy C (2006). Risks of contraception and pregnancy in heart disease. Heart.

[9] Abbas AE, Lester SJ, Connolly H (2005). Pregnancy and the cardiovascular system. Int J Cardiol.

[10] Lausman A, Kingdom J, Gagnon R, Basso M, Bos H, Crane J, Davies G, Delisle MF, Hudon L, Menticoglou S, Mundle W, Ouellet A, Pressey T, Pylypjuk C, Maternal Fetal Medicine Committee (2013). Intrauterine growth restriction: screening, diagnosis, and management. J Obstet Gynaecol Can.

[11] Kiely DG, Condliffe R, Webster V, Mills GH, Wrench I, Gandhi SV, Selby K, Armstrong IJ, Martin L, Howarth ES, Bu’lock FA, Stewart P, Elliot CA (2010). Improved survival in pregnancy and pulmonary hypertension using a multiprofessional approach. BJOG.

[12] Bassily-Marcus AM, Yuan C, Oropello J, Manasia A, Kohli-Seth R, Benjamin E (2012). Pulmonary Hypertension in Pregnancy: Critical Care Management. Pulm Med.

[13] Rao S, Ginns JN (2014). Adult congenital heart disease and pregnancy. Semin Perinatol.

[14] Weiss BM, Hess OM (2000). Pulmonary vascular disease and pregnancy: current controversies, management strategies, and perspectives. Eur Heart J.

[15] Bonnin M, Mercier FJ, Sitbo O, Roger-Christoph S, Jaïs X, Humbert M, Audibert F, Frydman R, Simonneau G, Benhamou D (2005). Severe pulmonary hypertension during pregnancy: mode of delivery and anesthetic management of 15consecutive cases. Anesthesiology.

[16] Wang H, Zhang WY, Liu T (2011). Experience of managing pregnant women with Eisenmenger’s syndrome: Maternal and fetal outcome in 13 cases. J Obstet Gynaecol Res.

[17] Curry R, Fletcher C, Gelson E, Gatzoulis MA, Woolnough M, Richards N, Swan L, Steer PJ, Johnson MR (2012). Pulmonary hypertension and pregnancy-a review of 12 pregnancies in nine women. B J O G.

[18] Subbaiah M, Kumar S, Roy KK, Sharma JB, Singh N (2013). Pregnancy outcome in women with pulmonary arterial hypertension: single-center experience from India. Arch Gynecol Obstet.

[19] Gurumurthy T, Hegde R, Mohandas BS (2012). Anaesthesia for a patient with Eisenmenger’s syndrome undergoing caesarean section. Indian J Anaesth.

[20] Ouyang DW, Khairy P, Fernandes SM, Landzberg MJ, Economy KE (2010). Obstetric outcomes in pregnant women with congenital heart disease. Int J Cardiol.

[21] Lin JH, Zhao WX, Su Y, Shi J, Jiang GJ, Wu ZM (2006). Perinatal management and pregnancy outcome in pregnant women with pulmonary hypertension complicating cardiac disease. Zhonghua Fu Chan Ke Za Zhi.

[22] Drenthen W, Pieper PG, Roos-Hesselink JW, van Lottum WA, Voors AA, Mulder BJ, van Dijk AP, Vliegen HW, Yap SC, Moons P, Ebels T, van Veldhuisen DJ (2007). Outcome of pregnancy in women with congenital heart disease: a literature review. J Am Coll Cardiol.

[23] Stoddart P, OSullivan G (1993). Eisenmenger’s Syndrome in Pregnancy: A case report and review. Internat J Obstet Anesth.

[24] Phupong V, Ultchaswadi P, Charakorn C, Prammanee K, Prasertsri S, Charuluxananan S (2003). Fatal maternal outcome of a parturient with Eisenmenger’s syndrome and severe preeclampsia. Arch Gynecol Obstet.

[25] Wang H, Liu T (2013). Pregnancy and Hemolysis, Elevated Liver Enzymes and Low Platelet Count Syndrome in Patients with Eisenmenger’s Syndrome. Am J Med Sci.

